# A cationic lumen in the Wzx flippase mediates anionic O-antigen subunit translocation in *Pseudomonas aeruginosa* PA01

**DOI:** 10.1111/j.1365-2958.2012.08084.x

**Published:** 2012-05-23

**Authors:** Salim T Islam, Robert J Fieldhouse, Erin M Anderson, Véronique L Taylor, Robert A B Keates, Robert C Ford, Joseph S Lam

**Affiliations:** 1 Department of Molecular and Cellular BiologyGuelph, ON, N1G 2W1, Canada; 2 Biophysics Interdepartmental Group, University of GuelphGuelph, ON, N1G 2W1, Canada; 3 Faculty of Life Science, University of ManchesterManchester M60 1QD, UK

## Abstract

Heteropolymeric B-band O-antigen (O-Ag) biosynthesis in *Pseudomonas aeruginosa* PAO1 follows the Wzy-dependent pathway, beginning with translocation of undecaprenyl pyrophosphate-linked anionic O-Ag subunits (O units) from the inner to the outer leaflets of the inner membrane (IM). This translocation is mediated by the integral IM flippase Wzx. Through experimentally based and unbiased topological mapping, our group previously observed that Wzx possesses many charged and aromatic amino acid residues within its 12 transmembrane segments (TMS). Herein, site-directed mutagenesis targeting 102 residues was carried out on the TMS and loops of Wzx, followed by assessment of each construct's ability to restore B-band O-Ag production, identifying eight residues important for flippase function. The importance of various charged and aromatic residues was highlighted, predominantly within the TMS of the protein, revealing functional ‘hotspots’ within the flippase, particularly within TMS2 and TMS8. Construction of a tertiary structure homology model for Wzx indicated that TMS2 and TMS8 line a central cationic lumen. This is the first report to describe a charged flippase lumen for mediating anionic O-unit translocation across the hydrophobic IM.

## Introduction

*Pseudomonas aeruginosa* is an opportunistic Gram-negative bacterial pathogen that often infects individuals with compromised defences resulting from such conditions as AIDS, cancer, severe burn wounds or cystic fibrosis (Lyczak *et al*., 2000). Lipopolysaccharide (LPS) in the outer leaflet of the outer membrane of the bacterium is a key virulence factor that can also affect a range of other virulence traits such as type III effector secretion, flagellar motility, type IV pilus action and biofilm formation (Lam *et al*., 2011).

Lipopolysaccharide is a glycolipid composed of three distinct domains, namely the endotoxic lipid A moiety, the core oligosaccharide and the distal O-antigen (O-Ag) capping motif. *P. aeruginosa* PAO1 synthesizes both a homopolymeric common polysaccharide antigen (A band) and a heteropolymeric O-specific (B band) O-Ag glycoform, with the latter composed of repeating trisaccharide O units each containing a proximal d-fucosamine sugar followed by two dideoxy-mannuronic acid derivatives (Knirel *et al*., 2006). B-band O-Ag is the immunodominant cell-surface antigen in *P. aeruginosa*; thus, variations in its composition and structure are responsible for classification of the bacterium into 20 distinct serotypes (Lam *et al*., 2011).

B-band O-Ag is synthesized via the Wzy-dependent assembly pathway, requiring the sequential action of four integral inner membrane (IM) proteins believed to form a complex (Whitfield, 1995). Following cytoplasmic synthesis of the trisaccharide repeat unit on the lipid carrier molecule undecaprenyl pyrophosphate (UndPP), the flippase Wzx mediates translocation of these lipid-linked O-unit trisaccharides from the inner to the outer leaflet of the IM (Liu *et al*., 1996; Burrows and Lam, 1999). Polymerization of B-band repeats at the reducing terminus is carried out by Wzy (Robbins *et al*., 1967; de Kievit *et al*., 1995; Woodward *et al*., 2010) via a putative catch-and-release mechanism (Islam *et al*., 2011), to preferred modal lengths of 12–16 and 22–30 units regulated by Wzz_1_, or 40–50 units regulated by Wzz_2_ (Daniels *et al*., 2002). Finally, ligation of polymerized O-Ag to lipid A-core is carried out by the O-Ag ligase WaaL (Abeyrathne *et al*., 2005; Abeyrathne and Lam, 2007), producing the mature LPS glycoform that is transported to the surface of the bacterium via the Lpt suite of proteins (Silhavy *et al*., 2010).

Wzx flippases belong to the prokaryotic polysaccharide transporter (PST) protein family, which in turn is one of four members of the multidrug/oligosaccharidyl-lipid/polysaccharide (MOP) exporter superfamily (Hvorup *et al*., 2003) (Fig. S1). Inter-family comparisons between the four MOP superfamily members indicate that the other three are more closely related to the PST family than to each other, suggesting that progenitors of the PST family were the evolutionary ancestors from which all MOP superfamily members arose. Consequently, a common mechanism of function may exist among them despite different substrate specificities (Hvorup *et al*., 2003).

Experimentally-based evidence to explain the potential mechanism of Wzx function had been lacking until a recent investigation in which we mapped the IM topologies of Wzx, Wzy and WaaL (Islam *et al*., 2010). An important observation regarding the 12 transmembrane segments (TMS) of Wzx from *P. aeruginosa* PAO1 (Wzx*_Pa_*) was the presence of numerous charged and aromatic amino acids in these domains compared with models based on topology prediction algorithms (Islam *et al*., 2010). Furthermore, data from the analysis of helical wheel diagrams corresponding to the residues within each TMS revealed distinct faces on certain α-helices containing charged, polar and aromatic amino acids (Islam *et al*., 2010).

Site-directed mutagenesis of 102 residue positions spanning the protein was performed to identify residues of functional importance for the flipping of UndPP-linked O units, as assayed by the ability of a construct to restore B-band LPS biosynthesis in a *wzx* chromosomal mutant (Burrows and Lam, 1999). Densitometric analysis was performed to quantify the change in B-band LPS production relative to wild-type protein complementation levels. Importantly, we identified numerous charged, polar and aromatic residues crucial for Wzx*_Pa_* function, many of which are present within the TMS. To further understand the structural context of these residues, we built a tertiary structure homology model for Wzx*_Pa_*, which revealed the presence of a cationic central channel lumen. Together, these findings provide the first tertiary structure evidence to strongly support the presence of a charged flippase lumen for accommodating anionic O-unit translocation across the hydrophobic IM by Wzx*_Pa_*.

## Results

### Charged and aromatic amino acids are required for O-unit translocation by Wzx*_Pa_*

Site-directed mutagenesis of Wzx*_Pa_* was carried out within the context of the experimentally-derived topology map of the protein (Fig. S2) and the helical-wheel representations of its TMS (Islam *et al*., 2010) to detect amino acids important for flippase function. Mutations were introduced in a previously-created fully-functional construct encoding Wzx fused C-terminally with a His_8_-tagged green fluorescent protein moiety (Wzx-GFP-His_8_) (Islam *et al*., 2010). Each construct was assayed for its ability to complement a *P. aeruginosa* PAO1 *wzx* knockout mutant and restore synthesis of B-band LPS. In total, 148 different substituted constructs were created representing 102 amino acid residue positions in the loops and the TMS regions, including all of the charged and many of the aromatic amino acid residues in these domains (Table S1).

**Fig. 1:**
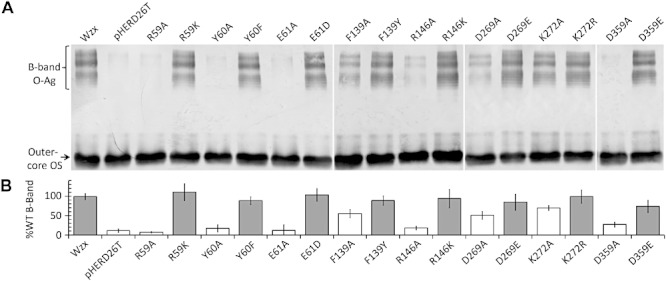
LPS analysis of *P. aeruginosa* PAO1 Δ*wzx* complemented with *wzx-gfp-his_8_* mutant constructs. A. Western immunoblot analysis of LPS from complemented strains. The blot was simultaneously probed with anti-B-band O-Ag mAb M15-4 (Lam *et al*., 1987) and anti-outer core oligosaccharide mAb 5C-101 (de Kievit and Lam, 1994). B. Densitometry analysis of the above Western immunoblots. The ratio of B-band O-Ag density to that of outer core oligosaccharide was compared between *P. aeruginosa* PAO1 Δ*wzx* complemented with the native *wzx-gfp-his_8_* construct versus the various mutants. Bars represent mean values of three biological replicates (*n* = 3) each measured in triplicate. Error bars are displayed ± standard error. Statistical significance of the density differences of each mutant compared with the native construct were calculated using the Student's *t*-test; mean value bars in white and grey display differences that are statistically significant (*p* ≤ 0.05) and not statistically significant (*p* > 0.05), respectively, compared with complementation with the native construct. Individual *p* values for the data are as follows: pHERD26T empty-vector control (0.0001), R59A (0.0001), R59K (0.9526), Y60A (0.0001), Y60F (0.4873), E61A (0.0001), E61D (0.8429), F139A (0.0031), F139Y (0.2456), R146A (0.0001), R146K (0.7718), D269A (0.0082), D269E (0.2722), K272A (0.0003), K272R (0.8670), D359A (0.0004), D359E (0.1506). Densitometry data and statistical analyses for all mutant constructs screened are available in *Supporting information* (Table S1).

Loss of aromatic properties via individual substitutions Y60A and F139A resulted in compromised abilities of each mutant protein to complement the Δ*wzx* mutant and restore B-band LPS biosynthesis (Fig. 1A). In each case, a significant reduction in B-band LPS production (*p* ≤ 0.05) was observed compared with the native construct (Fig. 1B). Maintenance of a benzyl-derived aromatic group at these respective positions via substitutions Y60F and F139Y was sufficient to restore B-band LPS production (Fig. 1A) to levels equivalent to the native construct (Fig. 1B), indicating the importance of benzyl aromatic groups at these amino acid positions.

Removal of charge characteristics via substitutions R59A, E61A, R146A, D269A, K272A and D359A also resulted in functionally-deficient Wzx*_Pa_* variants that displayed significant decreases in B-band LPS levels (*p* ≤ 0.05) in the complementation assay (Fig. 1). In contrast, ‘like charge’-substituted constructs R59K, E61D, R146K, D269E, K272R, and D359E maintained native levels of B-band LPS production (Fig. 1), demonstrating the requirement of the respective cationic or anionic charge at these positions for Wzx*_Pa_* function.

As position-dependent alteration of charge or aromatic characteristics has the potential to affect the membrane insertion of TMS (Gafvelin and von Heijne, 1994; Braun and von Heijne, 1999), the relative amounts of mutant constructs inserted in the membrane were compared with that of the native Wzx*_Pa_*-GFP-His_8_ construct. This was accomplished via analysis of GFP fluorescence levels from respective membrane preparations, indicating the various mutations did not abrogate membrane insertion (Fig. S3); this would suggest that native TMS packing events and hence stability of the constructs in the membrane was not affected.

### TMS2 and TMS8 line the channel lumen of a Wzx*_Pa_* tertiary structure homology model

**Fig. 2:**
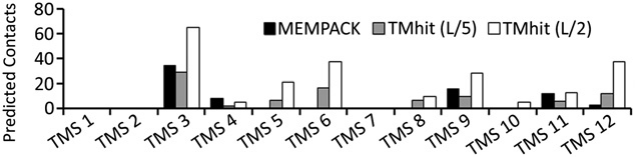
Support vector machine (SVM) contact prediction analysis between TMS of Wzx*_Pa_*. The number of total contacts (*y*-axis) for a given TMS (*x*-axis) are displayed based on MEMPACK (Nugent and Jones, 2010) output (black bars) as well as TM*hit* (Lo *et al*., 2009) output at both the high-threshold L/5 (grey bars) and low-threshold L/2 (white bars) settings.

Given the importance of TMS residues revealed via mutagenesis, the propensity for each membrane domain to form contacts with others was examined to gain insights into the manner in which they may pack relative to each other. This was accomplished using the MEMPACK (Nugent and Jones, 2010) and TM*hit* (Lo *et al*., 2009) support vector machine classification approaches, which minimize the over-fitting of data (Schneider and Fechner, 2004). These analyses indicated that TMS3, 4, 9, 11 and 12 possessed a high number of potential contacts, indicating that they are likely sequestered within helical bundles; this is in contrast to TMS1, 2, 7, 8 and 10 that displayed only a small number of predicted contacts, suggesting that they are not buried within such structures (Fig. 2).

**Fig. 3:**
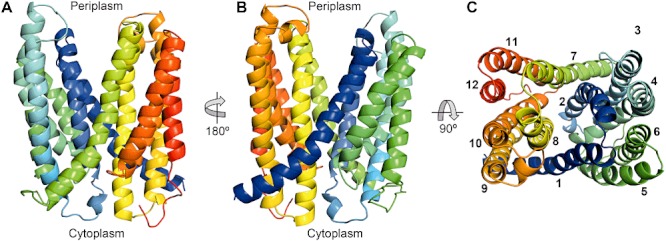
Tertiary structure homology model for Wzx*_Pa_*. The model was based on the X-ray crystal structure of the closely related protein NorM from *Vibrio cholerae* O1 El Tor (He *et al*., 2010). A and B. (A) Back view and (B) front view of Wzx*_Pa_*, with periplasmic and cytoplasmic sides indicated. C. Periplasmic view, with helices TMS1–TMS12 marked. The structure is coloured using an N-terminal (blue) to C-terminal (red) rainbow gradient.

To substantiate the MEMPACK and TM*hit* observations (Fig. 2), a tertiary structure homology model was constructed for Wzx*_Pa_* (Fig. 3) as outlined in *Experimental procedures* using established methodologies (Ravna and Sylte, 2012). This model was based on the recently-determined high-resolution X-ray crystal structure of NorM from *Vibrio cholera* O1 El Tor (NorM*_Vc_*; PDB ID: 3MKT) (He *et al*., 2010) and as such displays a twofold rotational symmetry (Fig. 3C). NorM is classified as a member of the multidrug and toxin extrusion (MATE) family of proteins, which pumps out drugs and other toxic compounds from the cytoplasm (Morita *et al*., 2000) consistent with other MATE family members (Huda *et al*., 2001; Chen *et al*., 2002). As with Wzx, NorM is a part of the MOP exporter protein superfamily (Hvorup *et al*., 2003) (Fig. S1). Wzx*_Pa_* displays notable sequence homology (Fig. S4A) and hydrophobicity profile similarity (Fig. S4B) to NorM*_Vc_*. In addition, the first half of the Wzx*_Pa_* primary structure (residues 1–206) aligns well with the second half of the protein (residues 207–411) (Fig. S5), a consistent trait of MATE family transporter proteins (Hvorup *et al*., 2003). Furthermore, the 3MKT structure was the top-ranked template (Probability = 99.9%; *E*-value = 1.4 × 10^−25^) in the HHpred fold-recognition search (Söding *et al*., 2005) when the amino acid sequence of Wzx*_Pa_* was used as a query for homologue detection. Taken together, these data provided further support for using NorM*_Vc_* as a template for building a structural homology model of Wzx*_Pa_* (Fig. 3).

The quality of the Wzx*_Pa_* structural model was assessed using genetic, biochemical and bioinformatic approaches, beginning with the sequence comparison of homologues. Residue pairs in homologous proteins that are distantly separated in primary structure but in direct contact via tertiary structure have a high propensity to maintain amino acids at these positions that have similar physicochemical and steric properties to match polarities or conserve molecular volume (Olmea and Valencia, 1997). The sequences of the 32 Wzx proteins were examined for such correlated pairs occurring at the contact nodes between TMS1–TMS8, TMS2–TMS7, TMS2–TMS8 and TMS3–TMS7 of the Wzx*_Pa_* structural model. These helix pairs represent the zones at which the N- and C-terminal halves of the protein interact (Fig. 3); such interactions would likely be important for conformational changes required to complete the transport mechanism (Wood *et al*., 2005; Schushan *et al*., 2012). Amino acids at specific positions in the primary structure were frequently found to maintain such pairs of residues at these interaction nodes in the tertiary structure of Wzx*_Pa_* (Table S2), consistent with their expected conservation.

**Fig. 4:**
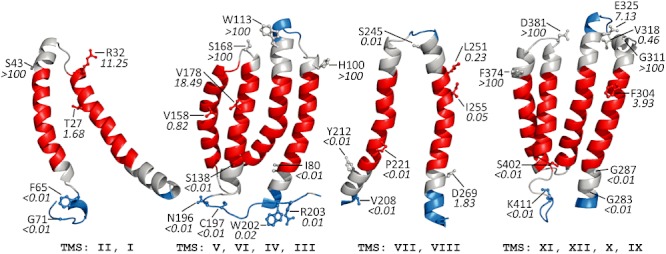
Wzx*_Pa_* homology model validation through truncation fusion to a PhoALacZα dual reporter. Model coloured according to output from iMembrane (Kelm *et al*., 2009) indicating predicted position of Wzx*_Pa_* within different lipid bilayer regions resulting from molecular dynamics simulations (red, lipid acyl chains; grey, phosphate headgroups; blue, exposed). Displayed residues are depicted with their corresponding normalized activity ratios (NARs) of PhoA activity to LacZ activity (Table S3) indicating cytoplasmic (NAR < 0.01), transmembrane (NAR 0.01–100) or periplasmic (NAR > 100) PhoALacZα dual reporter localization (Alexeyev and Winkler, 1999; Islam *et al*., 2010).

The location of loop and core TMS regions in the Wzx*_Pa_* model structure was further supported through the analysis of existing targeted and random C-terminal truncations of the flippase fused to a unique PhoALacZα dual reporter (Islam *et al*., 2010). This dual-reporter system displays high alkaline phosphatase (AP) activity and negligible β-galactosidase (BG) activity when expressed as a fusion localized to a periplasmic membrane protein domain. Alternatively, high BG activity (via α-complementation) and minimal AP activity is displayed upon expression as a cytoplasmic fusion (Alexeyev and Winkler, 1999). The capacity for two enzyme activities from the same reporter allowed for their comparison for each PhoALacZα fusion irrespective of fusion expression level as their stoichiometric ratios remained equivalent. Both AP and BG enzyme activities from various truncation fusion constructs were assayed and normalized against the highest reported activity of a given truncation set to obtain normalized activity ratios (NAR) (Table S3). NAR for the PhoALacZα fusions under investigation were displayed on a segmented model of Wzx*_Pa_* overlaid with output from iMembrane (Fig. 4), a program that predicts the position of a membrane protein within a lipid bilayer based on molecular dynamics simulations (Kelm *et al*., 2009). NAR of < 0.01, 0.01–100 and > 100 are representative of cytoplasmic, core TMS and periplasmic PhoALacZα dual reporter localizations respectively (Alexeyev and Winkler, 1999; Islam *et al*., 2010). Wzx*_Pa_* truncation fusions possessing these NAR were found to correspond well with peripheral cytoplasmic, core TMS and peripheral periplasmic domains predicted by iMembrane, respectively, further reinforcing the Wzx*_Pa_* structural model (Fig. 3). The orientation of Wzx*_Pa_* is also consistent with the previous observation of a fully-functional protein containing a fluorescent C-terminal fusion to GFP (Islam *et al*., 2010), a reporter that is only fluorescent in the cytoplasm (Aronson *et al*., 2011).

**Fig. 5:**
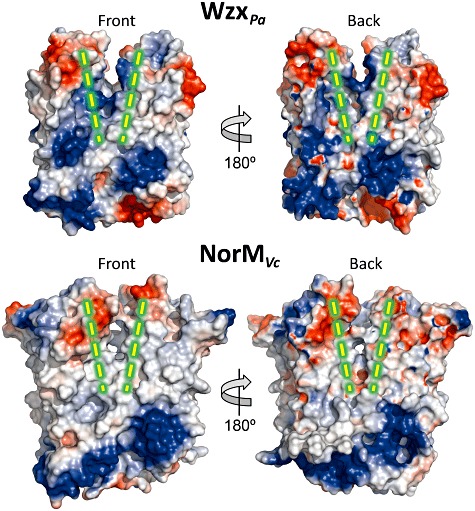
Surface electrostatic potential for the structural model of Wzx*_Pa_* (Fig. 3) and the structure of NorM*_Vc_* as originally published (He *et al*., 2010). The portals formed by TMS1 and TMS8 (front) as well as TMS2 and TMS7 (back) that open to the outer leaflet of the IM are marked with dashed yellow-and-green lines. Protein surfaces have been coloured according to residue charge, from blue (positive) to red (negative). Hydrophobic/uncharged surfaces have been coloured in white.

Consistent surface electrostatic properties were also observed when the NorM*_Vc_* and Wzx*_Pa_* structures were compared (Fig. 5); the only difference was the presence of positive charge lining the front portal of Wzx*_Pa_*, a feature not seen in NorM*_Vc_* (Fig. 5). Positive charge equivalent to that seen in NorM*_Vc_* was revealed on the cytoplasmic face of Wzx*_Pa_*, consistent with the ‘positive-inside rule’ demonstrated in membrane protein structures (Ulmschneider *et al*., 2005). The quality of the final Wzx*_Pa_* homology model was also evaluated with both the Meta Model Quality Assessment Program II (MetaMQAP II) (Pawlowski *et al*., 2008) and MolProbity (Chen *et al*., 2010); the former indicated a high degree of model quality (Fig. S6A), while the latter indicated the satisfaction of conformational restraints by the residues of the Wzx*_Pa_* model (Fig. S6B).

### The lumen of Wzx*_Pa_* forms a positively-charged channel

**Fig. 6:**
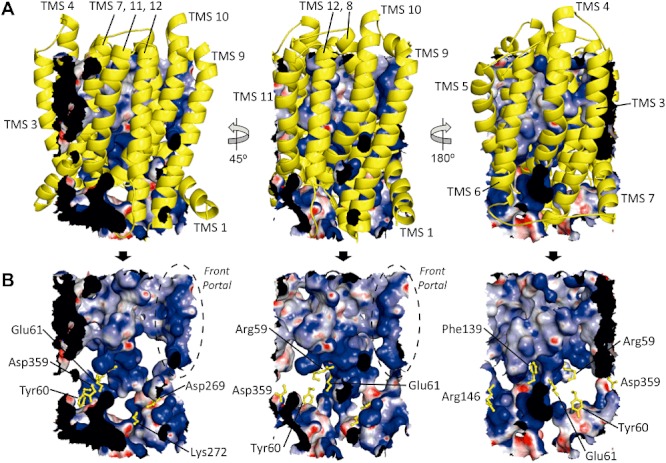
Characteristics of the Wzx*_Pa_* lumen. Internal volume was determined using HOLLOW (Ho and Gruswitz, 2008) and overlaid with the electrostatic potential of the channel interior. Surfaces have been coloured according to charge, from blue (positive, +15 kT/e) to white (uncharged/hydrophobic), to red (negative, −15 kT/e). A. Backbone structure of Wzx*_Pa_* (Fig. 3) with relevant TMS labelled (yellow), overlaid on the HOLLOW structure, to indicate the position of the internal cavity within Wzx*_Pa_*. B. HOLLOW output depicting the interior void volume of Wzx*_Pa_*, with the protein backbone structure removed for clarity. Amino acids with demonstrated functional importance (Fig. 1) are indicated in yellow. The region of interior volume corresponding to the front periplasmic exit portal of Wzx*_Pa_* (Fig. 5) is indicated by a dashed oval.

The lumenal volume of the Wzx*_Pa_* structure was determined through filling of the internal void space with dummy atoms using HOLLOW (Ho and Gruswitz, 2008) (Fig. 6A); these results revealed a substantial central chamber (Fig. 6B). Calculation of the overall electrostatic potential of this surface contributed by the amino acid side chains lining the lumen indicated overwhelmingly-cationic charge properties within the centre of the channel (Fig. 6B) as well as in the top half of the protein (Fig. 6B, middle panel) corresponding to the front periplasmic portal (Figs 5 and 6B); this was further reinforced by an overall lack of anionic (red) or uncharged/hydrophobic (white) colouration on the HOLLOW output (Fig. 6B). While NorM*_Vc_* was expectedly revealed to contain an internal chamber, it was not found to be heavily charged (Fig. S7), unlike that of Wzx*_Pa_* (Fig. 6). All identified residues of functional importance (Fig. 1) were found to be present within the cytoplasmic half of Wzx*_Pa_*; several (Arg59, Tyr60, Glu61, Phe139 and Lys272) were found to be in direct contact with the lumen, whereas others (Arg146, Asp269 and Asp359) were partially buried within the tertiary structure in the current conformation of the flippase (Fig. 6B).

## Discussion

The Wzy-dependent assembly pathway is conserved in a wide range of Gram-negative and Gram-positive bacteria for the synthesis of various cell-surface glycans (Raetz and Whitfield, 2002; Cuthbertson *et al*., 2009); in many of the former, it is responsible for heteropolymeric O-Ag biosynthesis. Once the precursor oligosaccharide O units have been synthesized at the cytoplasmic leaflet of the IM on the lipid carrier UndPP, the first proposed assembly step involves translocation of these lipid-linked O units across the IM to its outer leaflet (Whitfield, 1995). This represents a thermodynamically unfavourable scenario for LPS biosynthesis in *P. aeruginosa* PAO1, as it requires the translocation of a substantially anionic UndPP-linked trisaccharide substrate across the hydrophobic IM lipid bilayer.

The presence of such an unusually high number of charged TMS amino acid residues as those uncovered in Wzx*_Pa_* (Islam *et al*., 2010) is a phenomenon that had not been observed in topological studies of O-unit flippases in other bacteria (Mazur *et al*., 2005; Cunneen and Reeves, 2008; Marolda *et al*., 2011). The charged amino acids in several of the TMS of Wzx*_Pa_* were also found to localize to distinct helical faces of the TMS. These results formed the basis for proposing a mechanism of Wzx*_Pa_* function, which is that it possesses a charged lumen to mediate translocation of UndPP-linked anionic O units across the IM (Islam *et al*., 2010).

In this study we have presented the findings of the most extensive and systematic mutagenesis screen of a MOP exporter superfamily protein (Fig. S1). Consequently, we have demonstrated the functional importance of multiple charged and aromatic residues within the context of a rigorously-constructed tertiary structure homology model built for Wzx*_Pa_*, indicating the presence of a cationic channel lumen in the flippase. These results reinforce the mechanism of Wzx*_Pa_* function first proposed by our group (Islam *et al*., 2010).

Wzx and NorM are both members of the MOP exporter superfamily, a group that also includes the Rft1 protein, originally classified as part of the oligosaccharidyl-lipid flippase (OLF) family (Hvorup *et al*., 2003) (Fig. S1), which has been shown to play a role *in vivo* in the translocation of a dolichol pyrophosphate-linked heptasaccharide unit from the cytoplasmic to the lumenal leaflet of the endoplasmic reticulum membrane in eukaryotes (Helenius *et al*., 2002; Frank *et al*., 2008; Rush *et al*., 2009). Given the relatedness between NorM*_Vc_* and Wzx*_Pa_* (Figs S1, S4 and S5), it was appropriate to use NorM*_Vc_* as a template for modelling the structure of Wzx*_Pa_* through the satisfaction of spatial restraints via MODELLER (Eswar *et al*., 2007); this was accomplished using a similar approach to that used for the successful modelling of the *Escherichia coli* osmosensor and transporter ProP against the X-ray crystal structure of the lactose permease LacY (Wood *et al*., 2005; Liu *et al*., 2007). The NorM*_Vc_* structure was proposed to have been determined in the closed state of the protein in which the cytoplasmic face has not yet transitioned to a state favourable for substrate binding (He *et al*., 2010); by extension, the Wzx*_Pa_* structure would also reflect this characteristic. Additionally, the packing arrangement of TMS in the MOP exporter superfamily members NorM and Wzx is different from that present in LacY (Guan *et al*., 2007). LacY is a member of the major facilitator superfamily (Forrest *et al*., 2011) and was previously suggested to possess a TMS arrangement reflective of that proposed for Wzx proteins (Marolda *et al*., 2010; 2011).

The ability to substitute each of the eight functionally important residues identified in this study with amino acids possessing similar charges or aromatic characteristics suggests that the tertiary structure context in which these functional groups are located is the important functional determinant, rather than the specific stereochemistry contributed by each side chain. As aromatic residues are commonly located in the binding sites of sugar- and carbohydrate-binding proteins (Malik and Ahmad, 2007; Elumalai *et al*., 2010), this is consistent with the importance of Tyr60 and Phe139 for Wzx*_Pa_* function (Fig. 1). These residues may be involved in substrate binding during translocation as they are internally located within the protein (Fig. 6).

The cationic Arg59 and Lys272 as well as anionic Glu61 and Asp269 residues are all internally located in the Wzx*_Pa_* structure, suggesting their involvement in substrate translocation. Lumenal cationic residues could be important for interaction with the anionic substrate. Meanwhile, anionic residues may serve to ‘push’ the substrate through the lumen via charge repulsion from conformational changes during translocation. Residue Arg146 may play a role in initial recognition events prior to substrate translocation, as it is not entirely lumenal compared with the cationic residues mentioned above. Following O-unit translocation, the front cationic portal of Wzx*_Pa_* (Figs 5 and 6B) would provide a likely site of lateral exit for the UndPP-linked O-Ag into the periplasmic leaflet of the IM, ready for subsequent polymerization by Wzy. The involvement of the UndPP lipid carrier in translocation is not yet known, but we speculate that it could partially enter the Wzx molecule between TMS1 and TMS9 (Fig. 3C) in such a way that the acyl chain remains embedded in the IM (Zhou and Troy, 2005) while the pyrophosphate-linked O unit travels through the lumen of Wzx. This would entail that the exit of the O unit should also happen from between TMS1 and TMS9, which corresponds to the front periplasmic portal (Fig. 6B).

The Arg59–Tyr60–Glu61 residue tract is essential for flippase function (Fig. 1) and highly conserved among the 31 blastp hits against Wzx*_Pa_* (Table S2), despite the overall lack of high sequence identity and the expected substrate specificity differences. As the mechanism of O-unit flipping would be expected to be conserved in this family of proteins, this may alternatively indicate a mechanistic role for these residues, possibly for proper loading of the substrate into the channel due to their central cytoplasmic localization. In general, transport proteins are widely recognized to undergo conformational changes during their respective transport cycles (Forrest *et al*., 2011; Henzler-Wildman, 2012); as such, residues that appear to be partially buried in the closed conformation of the protein are capable of becoming lumenally exposed during subsequent phases of the transport cycle (Forrest and Rudnick, 2009; Schushan *et al*., 2012).

Charge-dependent substitutions D85A, R298A, D326A and K419S have also been shown to be important for function in Wzx from *E. coli* O157:H7 (Wzx*_Ec_*) (Marolda *et al*., 2010; 2011). However, these residues do not align directly with any of the functionally-important residues identified in Wzx*_Pa_* (Fig. 1). While Arg298 and Asp326 in Wzx*_Ec_* are predicted to be periplasmic and cytoplasmic, respectively, Asp85 and Lys419 fall outside the limited region of the protein that was subjected to topology mapping via experimentation and as such their predicted localizations cannot be readily compared with the Wzx*_Pa_* structure (Fig. 3).

Intriguingly, all of the functionally-important residues identified in this investigation occur within the proximal (cytoplasmic) half of the protein, even though many mutants were made that map to the distal (periplasmic) half (Table S1). Despite its considerable size, cytoplasmic loop 3 (CL3) appears to serve primarily as a disordered linker peptide connecting TMS6 and TMS7 (Fig. 3), as numerous Ala substitutions among CL3 residues did not affect protein function (Table S1). Together, these observations suggest that the crucial domains involved in O-unit flipping act mainly during the initial binding phase to retain the substrate, and that the final extrusion step does not involve many stereospecific interactions once the lipid-linked substrate has reached the periplasmic half of the protein and is ready to be extruded.

For Wzx O-unit flippases, the long-held view has been that substrate specificity is dependent on direct recognition of the proximal UndPP-linked sugar moiety, irrespective of the distal sugar residues in the repeat unit (Marolda *et al*., 2004), and that it alone is sufficient for translocation (Feldman *et al*., 1999). However, recent elegant investigations involving the plant pathogens *Pantoea stewartii* (Stewart's wilt disease) and *Erwinia amylovora* (fire blight disease) (Wang *et al*., 2012), as well as the enteric *Salmonella enterica* groups B, D2 and E (Hong *et al*., 2012), have independently revealed that the various encoded Wzx proteins specifically transport substrates that contain identical UndPP-linked main-chain sugar units for their respective systems; the only difference is in the presence or absence of a capping sugar on a terminal side-branch decoration. These investigations examined the biosynthesis of exopolysaccharide (Wang *et al*., 2012) and O-Ag (Hong *et al*., 2012) respectively. From the cationic lumen of Wzx*_Pa_* revealed in our investigation (Fig. 6), it is conceivable that it would have arisen to accommodate the translocation of the two negatively charged terminal sugars of the *P. aeruginosa* PAO1 B-band O unit, as the proximal UndPP-linked sugar is neutral (Lam *et al*., 2011). The requirement for a positively-charged constriction to mediate transit of sugar polymers containing mannuronic acid has also been demonstrated in the recent X-ray crystal structure of AlgE, an outer-membrane beta-barrel protein required for the secretion of the anionic polymer alginate in *P. aeruginosa* (Whitney *et al*., 2011). Together, these findings suggest that deciphering the substrate specificity of Wzx proteins may be a more complex challenge than initially thought.

Several MATE family exporters have been shown to function via H^+^- or Na^+^-coupled antiport for the efflux of drug substrates from the cytoplasm (Morita *et al*., 2000; Huda *et al*., 2001; Chen *et al*., 2002). Consistent with these data, a Rb^+^ ion (Na^+^ structural analogue) was successfully co-crystallized with NorM*_Vc_*, leading He *et al*. to propose a mechanism for NorM*_Vc_* function (He *et al*., 2010). In accordance with this preliminary model for NorM*_Vc_* function, as well as the potential for a similar mechanism of function between MATE and PST proteins (Hvorup *et al*., 2003) (Fig. S1), and based on the structural model of Wzx*_Pa_* (Fig. 3) we propose that the O-unit flippase functions via a similar antiport mechanism. The essential Glu61, Asp269 and Asp359 carboxylates of Wzx*_Pa_* may be analogous to those of Asp36, Glu255 and Asp371 in NorM*_Vc_*, which in the latter may bind the antiported ion in the outward-facing conformation, and cationic substrates in the inward-facing conformation (van Veen, 2010).

This is in contrast to the results published earlier by Rick *et al*. in which the WzxE protein from *E. coli* K-12, required for synthesis of enterobacterial common antigen, was implicated in transport of a water-soluble nerol pyrophosphate (soluble UndPP analogue)-linked GlcNAc residue via simple diffusion (Rick *et al*., 2003). The shortcoming in this investigation stems from the authors' creation of vesicles from the IM; as such the role of other transport proteins in the IM in the rapid equilibration of substrate cannot be ruled out (Whitfield, 2006). To investigate this topic, biophysical studies are required to examine the gating stimuli of Wzx.

In conclusion, this study has provided the first structural data to help characterize the translocation process of UndPP-linked sugar substrate required for heteropolymeric LPS biosynthesis. As such, it represents a tertiary structure framework on which to base testable functional hypotheses and it presents a context in which to understand the function of such a widely conserved yet poorly understood protein.

## Experimental procedures

### DNA manipulations

Site-directed mutagenesis was carried out as previously described (Islam *et al*., 2011) on a plasmid template encoding Wzx fused with a C-terminal His_8_-tagged GFP moiety (Wzx-GFP-His_8_) previously shown to not impede protein function (Islam *et al*., 2010). The oligonucleotide mutagenesis primer sequences have been provided in the *Supporting information* (Table S4).

### LPS complementation analysis

The *in vivo* function of each mutant construct was assayed as previously described (Islam *et al*., 2011) in a *P. aeruginosa* PAO1 *wzx* chromosomal knockout mutant created by our group; this mutant was previously shown to be deficient in B-band LPS production while maintaining the production of A-band LPS (Burrows and Lam, 1999). LPS samples (3 µl) were analysed by SDS-PAGE and Western immunoblotting as previously described (Islam *et al*., 2011) with anti-B-band O-Ag and anti-outer core oligosaccharide mouse monoclonal antibodies (mAb) MF15-4 (Lam *et al*., 1987) and 5C-101 (de Kievit and Lam, 1994) respectively. Developed Western blots were scanned on a Bio-Rad GS-800 densitometer at 42.3 µm^2^ resolution. Quantification was performed using Quantity One software (Version 4.6.1) with linear regression and local background subtraction. The ratio of the density of the B-band O-Ag banding to that of the outer core oligosaccharide was compared between cells of *P. aeruginosa* PAO1 Δ*wzx* complemented with the native *wzx-gfp-his_8_* construct versus the various mutant constructs. Initial screens were performed using three independent singly-analysed samples, with statistical significance calculated in comparison with the native construct using the Student's *t*-test. Mutant constructs displaying densitometric complementation differences compared with the native construct but with non-statistically significant initial *t*-test results (due to high variability in the density ratio comparisons) were further examined through the use of cultures from three independently isolated clones (*n* = 3), each analysed in triplicate and averaged, before comparison with the native construct via the Student's *t*-test (Table S1). Membrane insertion of the various mutant constructs (Fig. S3) was carried out as previously described (Islam *et al*., 2011).

### Structure prediction and comparative modelling

MEMPACK (Nugent and Jones, 2010) was installed locally and run using the Ontario SHARCNET (Shared Hierarchical Academic Research Computing Network) system, with the required input psi-blast profile generated using the non-redundant protein database from NCBI. TM*hit* (Lo *et al*., 2009) was run online (http://bio-cluster.iis.sinica.edu.tw/TMhit/) using both the higher-threshold L/5 and lower-threshold L/2 settings. Model restraints for each program were introduced based on established Wzx core TMS boundaries (Islam *et al*., 2010).

Comparative modelling (Ravna and Sylte, 2012) was carried out in a similar fashion to that previously described for the successful generation of a homology model for the 12-TMS transporter ProP from *E. coli* (Wood *et al*., 2005; Liu *et al*., 2007), with several modifications. blastp analysis of the Wzx*_Pa_* amino acid sequence (GenBank Locus: NP_251843) was used to identify 31 prokaryotic Wzx homologues with minimal alignment gaps possessing 22–35% sequence identity (43–60% similarity). MUSCLE was used to generate a multiple sequence alignment (MSA) (Edgar, 2004) to identify positions that would tolerate sequence variability. The same procedure was followed for the amino acid sequence of NorM*_Vc_* (GenBank Locus: AE003852) to identify 43 NorM homologues possessing 53–68% sequence identity (72–85% similarity). MSAs of Wzx*_Pa_* and NorM*_Vc_*, respectively, with other homologues were aligned via MUSCLE to verify the alignment of stringent positions between the two sets of proteins and then compared using AlignMe (Khafizov *et al*., 2010) to verify the conserved nature of the respective hydrophobicity profiles (Fig. S4C). Results of coarse-grained molecular dynamics simulations of protein X-ray structures simulated in the presence of self-assembling membrane lipid bilayers, projected onto the NorM*_Vc_* structure using iMembrane (Kelm *et al*., 2009), allowed for the identification of the TMS core regions of NorM*_Vc_* (i.e. those in the same plane as the membrane lipid acyl chains). The TMS core regions of Wzx*_Pa_* and its 31 homologues were analysed with MEMSAT3 (Jones, 2007), yielding remarkably conserved positioning results when overlaid on the Wzx MSA. In conjunction with the topology map of Wzx*_Pa_* (Islam *et al*., 2010), this MSA provided a basis with which to assign residues in Wzx*_Pa_* to the exact TMS regions of NorM*_Vc_*. Wzx amino acid sequences corresponding to core TMS regions were manually aligned with their equivalents in NorM*_Vc_* such that no alignment gaps were present in these regions; special consideration was given to the demonstrated enrichment of the aromatic amino acids Tyr, Trp and His at the membrane interface regions of TMS in existing membrane protein crystal structures (Ulmschneider *et al*., 2005). This NorM*_Vc_*–Wzx*_Pa_* profile–profile alignment was used to generate 1000 homology models against the existing NorM*_Vc_* structure (PDB ID: 3MKT) (He *et al*., 2010) via molecular dynamics using MODELLER (Eswar *et al*., 2007), which was installed and run on SHARCNET under a 64-bit Fedora 14 architecture. Models were subjected to very-thorough molecular dynamics refinement in MODELLER, with that displaying the best discrete optimized protein energy (DOPE) score ultimately selected (Eswar *et al*., 2007). The quality of the final Wzx*_Pa_* homology model was evaluated with both MetaMQAP II (Pawlowski *et al*., 2008) and MolProbity (Chen *et al*., 2010). Surface electrostatics were displayed in PyMol for both Wzx*_Pa_* and NorM*_Vc_* to reflect the original data (He *et al*., 2010). Quantification of PhoA and LacZ activities for fusion constructs was carried out as previously described (Islam *et al*., 2010). The internal Wzx*_Pa_* chamber volume was analysed using HOLLOW (Ho and Gruswitz, 2008) with the overlaid solvent-accessible electrostatic potential calculated via APBS (Baker *et al*., 2001). All structure visualizations were generated using PyMol.
