# Supplementary material for: A cationic lumen in the Wzx flippase mediates anionic O-antigen subunit translocation in Pseudomonas aeruginosa PA01
Source: Mol Microbiol. 2012 May 23;84(6):1165–76. doi: 10.1111/j.1365-2958.2012.08084.x (PMC3412221; doi:10.1111/j.1365-2958.2012.08084.x)
Supplement: Supplementary file 1 [file mmi0084-1165-SD1.pdf]

SUPPORTING INFORMATION

A cationic lumen in the Wzx flippase mediates anionic O-antigen subunit translocation in *Pseudomonas aeruginosa* PAO1

Salim T. Islam[1], Robert J. Fieldhouse[1,2], Erin M. Anderson[1], Véronique L. Taylor[1], Robert A. B. Keates[1], Robert C. Ford[3], and Joseph S. Lam[1]*

[1]Department of Molecular and Cellular Biology and [2]Biophysics Interdepartmental Group, University of Guelph, Guelph, ON, N1G 2W1, Canada
[3]Faculty of Life Science, University of Manchester, Manchester, M60 1QD, United Kingdom

*corresponding author

Email: jlam@uoguelph.ca
Telephone: 519-824-4120 ext. 53823
Fax: 519-837-1802

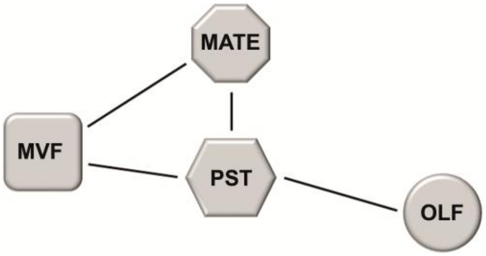

Figure S1. Schematic of the relative relatedness of the families of the MOP exporter superfamily. PST, polysaccharide transporter; MATE, multidrug and toxin extrusion; OLF, oligosaccharidyl-lipid flippase; MVF, mouse virulence factor (Hvorup *et al.*, 2003).

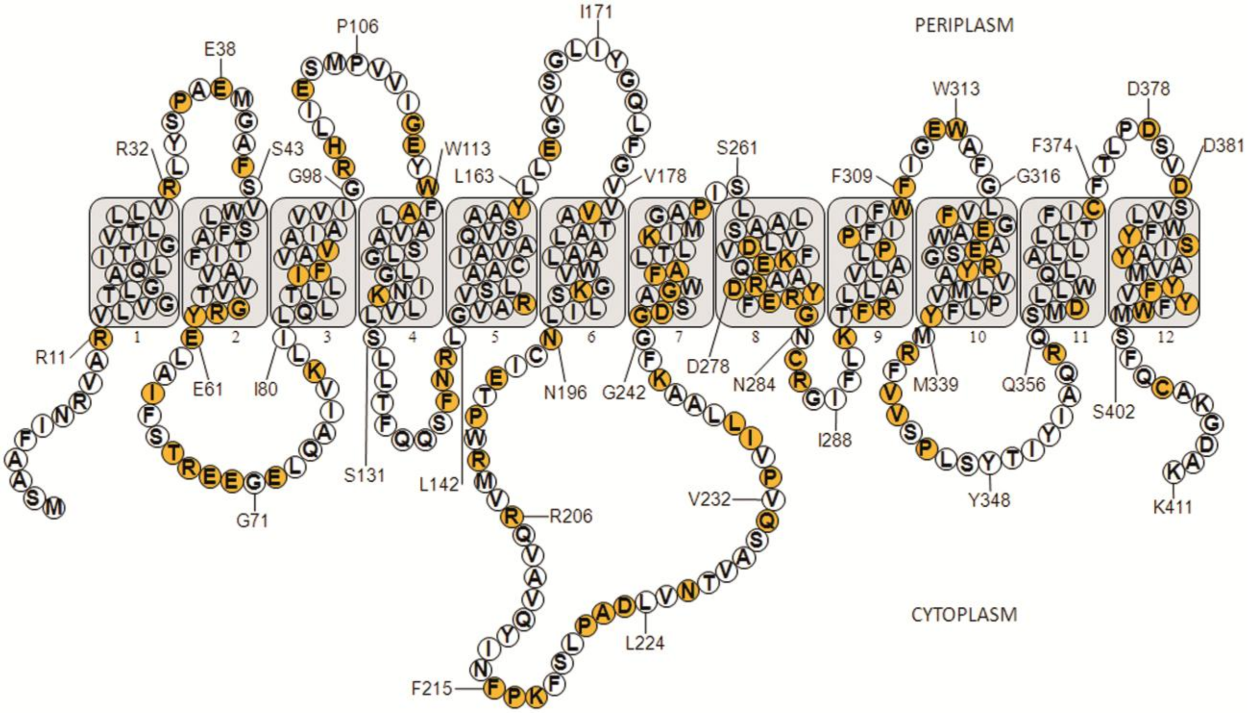

Figure S2. Locations of the 102 primary structure positions of Wzx (within the context of its experimentally-derived topology map (Islam *et al.*, 2010) targeted via site-directed mutagenesis to examine potential loss of function. TMS have been numbered from 1 – 12.

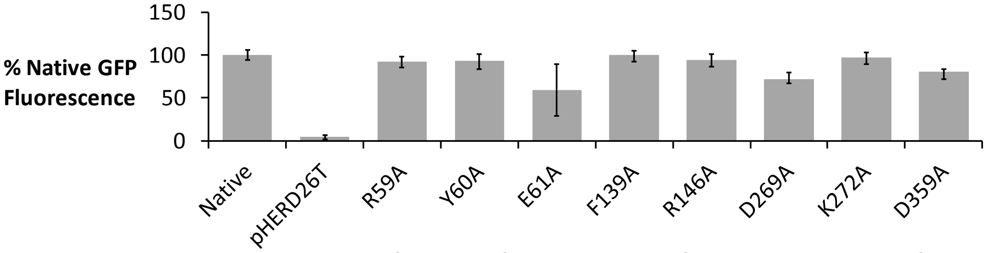

Figure S3. Densitometry analysis of GFP fluorescence from membrane fractions of *P. aeruginosa* PAO1 Δ*wzx* expressing native and mutant Wzx-GFP-His$_8$ constructs, carried out as previously described (Islam *et al.*, 2011). Fluorescence of Wzx-GFP-His$_8$ native and mutant constructs was analyzed in quadruplicate using ImageJ and displayed ± standard deviation.

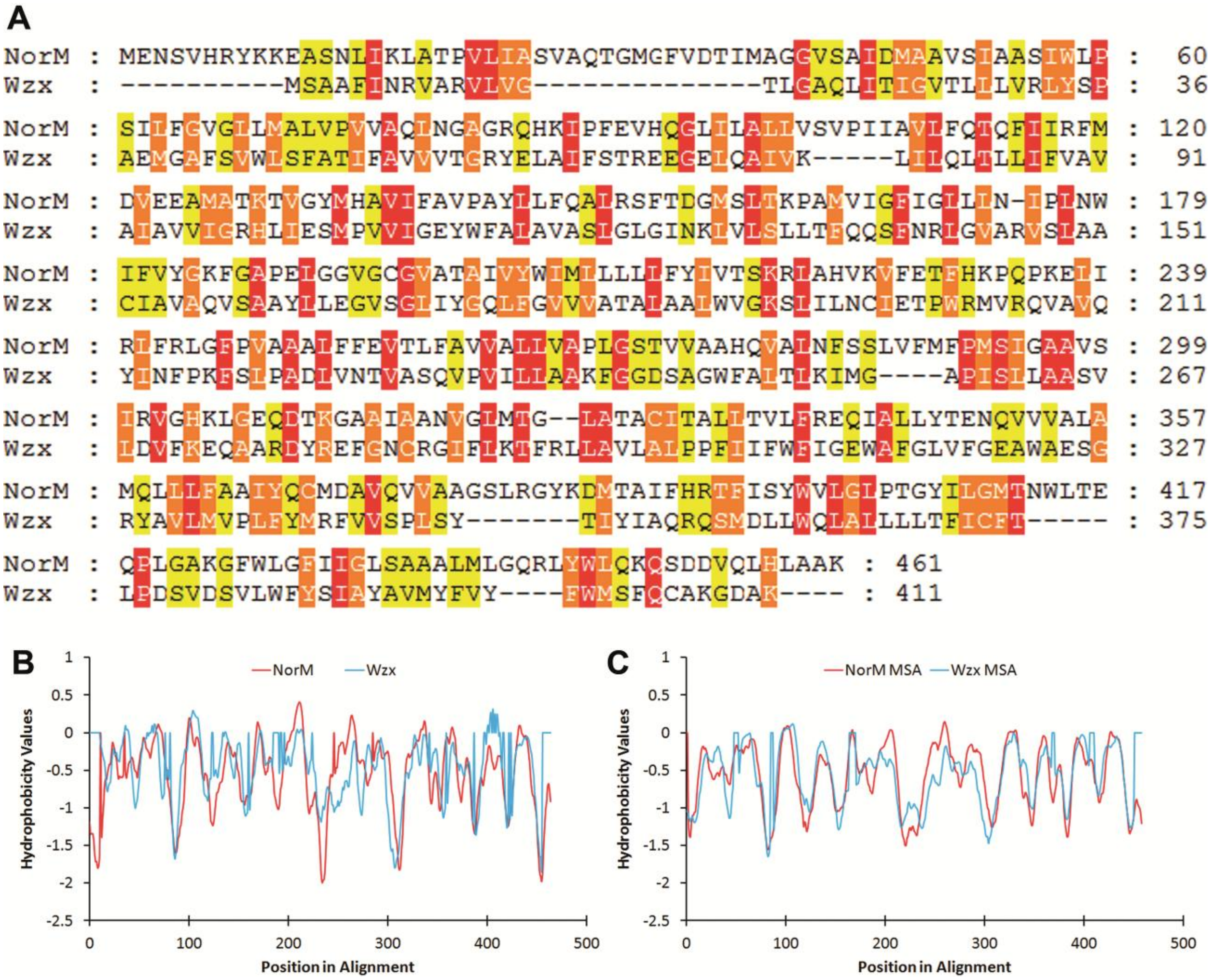

Figure S4. Comparison of NorM*Vc* and Wzx$_{Pa}$. **(A)** ClustalW2 amino acid sequence alignment. Residues have been coloured based on JalView (Waterhouse *et al.*, 2009) conservation scores (out of 10). Colour key: *red*, score = 10; *orange*, score = 9; *yellow*, score = 8. The two sequences were found to be 32.9% similar and 19.1% identical upon EMBOSS Stretcher pairwise alignment (matrix = BLOSUM62; gap penalty = 10, extend penalty = 2). **(B)** Hydropathy value comparison of NorM$_{Vc}$ (*red*) and Wzx$_{Pa}$ (*blue*) using AlignMe (Khafizov *et al.*, 2010). **(C)** Hydropathy value comparison between the multiple sequence alignments (MSA) comprised of BLASTp hits for NorM$_{Vc}$ (43 hits) (*red*) and Wzx$_{Pa}$ (31 hits) (*blue*) using AlignMe (Khafizov *et al.*, 2010). NorM MSA = 44 sequences in total (including NorM$_{Vc}$); Wzx MSA = 32 sequences in total (including Wzx$_{Pa}$).

```
Wzx_1-206           1 MSAA-FINRVARVLVGTLGAQLITIGVTLLLVRLYSPAEMGAFSVWLSF-   48
                      ..|. :||.....|...|...:.:....:||...:.....|.|::.|..
Wzx_207-411       207 QVAVQYINFPKFSLPADLVNTVASQVPVILLAAKFGGDSAGWFALTLKIM  256

Wzx_1-206          49 ----ATIFAVVVTGRYELAIFSTREEGELQAI-VKLILQLTLLIFVAVAI   93
                          :.:.|.|:....|.|....||.|..:.| :|....|.:|......|
Wzx_207-411       257 GAPISLLAASVLDVFKEQAARDYREFGNCRGIFLKTFRLLAVLALPPFII  306

Wzx_1-206          94 AVVIGRHLIESMPVVIGEYWFA---LAVASLGLGINKLVLSLLTFQQSFN  140
                      ...||..   :..:|.||.|..   .||..:.|...:.|:|.|    |:.
Wzx_207-411       307 FWFIGEW---AFGLVFGEAWAESGRYAVLMVPLFYMRFVVSPL----SYT  349

Wzx_1-206         141 RLGVARVSLAACIAVAQVSAAYLLEGVSGLIYGQLFGVVVATALAALWVG  190
                      .....|.|:.....:|.:...::...:...:...|:...:|.|:. .:|.
Wzx_207-411       350 IYIAQRQSMDLLWQLALLLLTFICFTLPDSVDSVLWFYSIAYAVM-YFVY  398

Wzx_1-206         191 KSLILNCIETPWRMVR  206
                      ..:...|.:..   .:
Wzx_207-411       399 FWMSFQCAKGD---AK  411
```

Figure S5. Pairwise alignment of Wzx residues 1–206 and 207–411 via EMBOSS Stretcher analysis (matrix = BLOSUM62; gap penalty = 12; extend penalty = 2). The two fragments were found to be 35.2% similar and 19.9% identical. **|** = an identical residue, **:** = a close conservative substitution, **.** = a more distant conservative substitution.

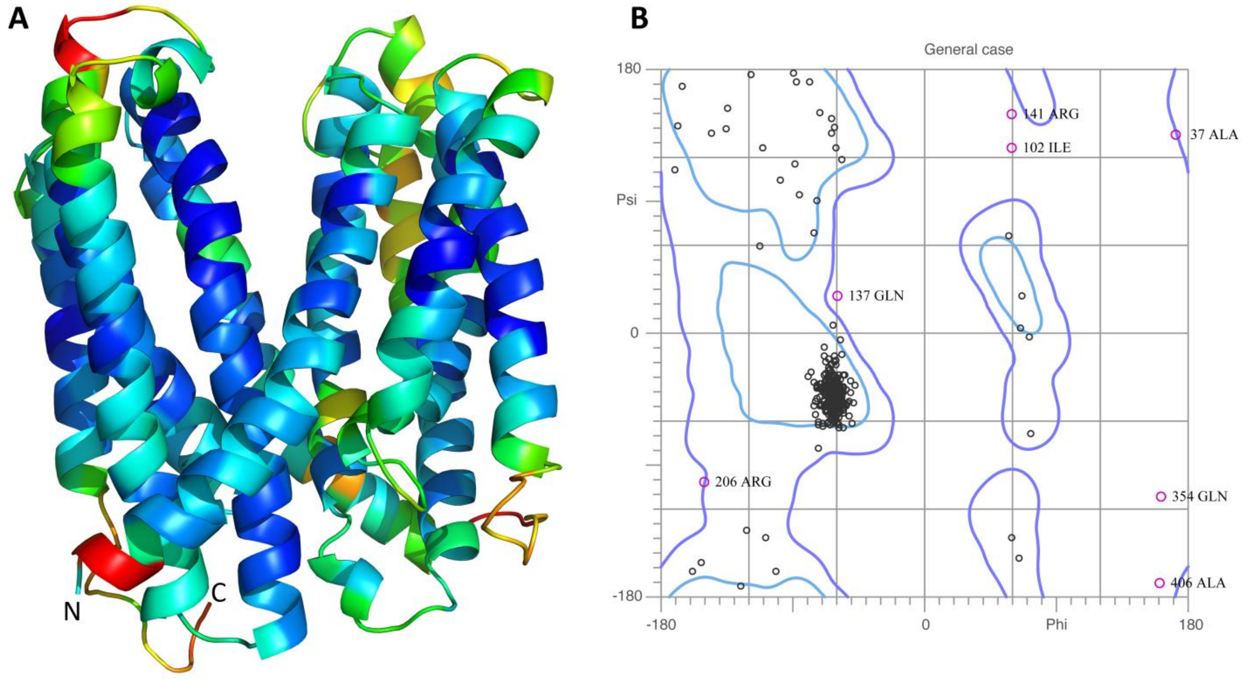

Figure S6. Wzx model quality assessment. **(A)** MetaMQAPII (Pawlowski *et al.*, 2008) overlay coloured with spectrum from higher quality (*blue*) to lower quality (*red*); Global Distance Test – Total Score (GDT–TS) = 42.092. **(B)** Ramachandran plot from MolProbity (Chen *et al.*, 2010) indicating the satisfaction of conformational restraints by the residues of the Wzx model (93.2% of all residues were in favoured [98%] regions, 98.0% of all residues were in allowed [>99.8%] regions).

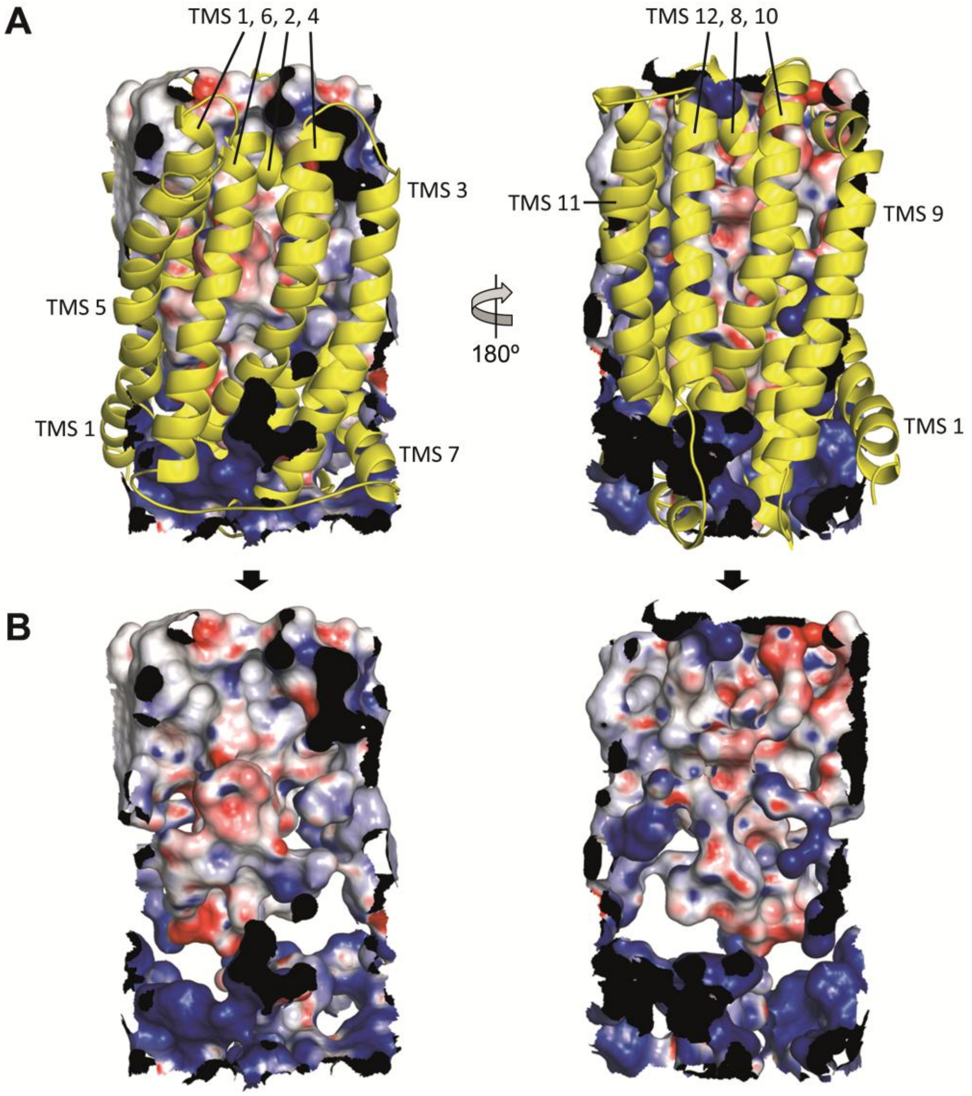

Figure S7. Characteristics of the NorM$_{Vc}$ lumen. **(A)** Structure of NorM$_{Vc}$ (He *et al.*, 2010) (PDB ID: 3MKT) overlaid with HOLLOW (Ho and Gruswitz, 2008) output indicating the void space within the NorM$_{Vc}$ structure. **(B)** HOLLOW output of NorM$_{Vc}$ with the corresponding protein backbone structure removed for clarity, overlaid with the electrostatic potential of the channel interior. Surfaces have been coloured according to charge, from *blue* (positive, +15 kT/e) to *white* (uncharged/hydrophobic) to *red* (negative, -15 kT/e). The same parameters were used for the HOLLOW output of Wzx$_{Pa}$ (Fig. 6).

Table S1. Densitometry data for B-band O-Ag complementation with various Wzx mutants

	Initial Screen[1]			*Follow-Up Screen*[2]		
Mutant	%WT	Std. Error (±)	T test	%WT	Std. Error (±)	T test
pHERD26T[3]	14.8	14.1	0.0059	11.0	4.3	0.0001
R11A	118.0	14.0	0.6457	118.0	14.3	0.1676
R32A	117.4	30.3	0.5296	–	–	–
P36A	108.9	30.6	0.8522	–	–	–
E38A	113.2	30.8	0.7787	–	–	–
F42A	109.8	13.0	0.7478	91.8	15.9	0.4600
G58V	126.2	11.4	0.2860	–	–	–
R59A	8.4	2.9	0.1102	6.9	1.8	0.0001
R59K	109.3	49.6	0.8434	110.5	21.5	0.9526
Y60A	19.1	2.5	0.0017	17.1	9.5	0.0001
Y60F	80.4	25.7	0.4732	88.3	10.0	0.4873
E61A	16.7	19.5	0.2025	11.9	14.1	0.0001
E61D	114.8	20.6	0.7692	103.4	16.3	0.8429
I64A	94.2	7.1	0.6640	99.6	9.3	0.8996
T67A	169.1	38.6	0.1980	–	–	–
R68A	104.4	6.7	0.4728	–	–	–
E69A	113.0	19.4	0.3779	–	–	–
E69A+E70A	104.3	14.4	0.8288	–	–	–
E69A+E70A+E72A	96.1	7.5	0.7357	130.7	43.0	0.3490
E70A	105.5	17.5	0.9532	–	–	–
E72A	94.4	10.2	0.6883	127.9	38.9	0.1978
K78A	101.3	1.2	0.4095	102.7	1.8	0.9845
K78R	101.4	0.8	0.1943	100.0	1.7	0.5243
I87A	145.5	53.8	0.2705	–	–	–
F88A	110.2	29.7	0.8731	106.1	25.2	0.8180
V91A	174.4	69.6	0.5082	–	–	–
R99A	96.7	2.8	0.5656	–	–	–
H100A	95.0	9.7	0.4156	–	–	–
E103A	129.0	35.6	0.9472	–	–	–
G110V	96.5	2.8	0.5799	108.8	41.2	0.7025
E111A	96.4	17.2	0.7724	95.9	12.8	0.6684
W113A	116.6	8.0	0.4494	127.0	23.5	0.1383
A115V	153.9	17.7	0.2016	–	–	–
K127A	79.6	7.3	0.5561	76.7	13.4	0.2091
F139A	65.6	24.3	0.2698	55.2	10.3	0.0031
F139Y	92.6	3.5	0.7708	88.7	12.0	0.2456
N140A	125.1	12.8	0.3142	–	–	–
R141A	102.6	14.0	0.6585	–	–	–
R146A	19.7	10.2	0.0561	17.9	4.6	0.0001
R146K	80.9	10.5	0.0823	94.1	23.4	0.7718
Y162A	76.5	15.7	0.4800	74.8	21.7	0.1150
E165A	101.9	38.0	0.9652	–	–	–
V180A	121.3	27.3	0.4632	–	–	–
K191A	128.4	68.8	0.4928	–	–	–
N196A	117.3	12.6	0.7421	–	–	–
E199A	85.9	5.8	0.7226	80.8	18.9	0.3281
P201A	112.0	26.2	0.7578	–	–	–
R203A	117.7	24.3	0.5336	–	–	–
R206A	101.8	1.7	0.8692	103.2	2.7	0.7414
R206K	99.7	15.2	0.8120	90.9	22.0	0.4559
F215A	85.3	7.4	0.3120	–	–	–
P216A	149.3	41.1	0.2446	–	–	–
K217A	103.5	24.5	0.8472	–	–	–
P221A	115.5	18.2	0.3965	–	–	–

Table S1. (ctd.)

A222S	105.9	4.6	0.5160	–	–	–
D223A	93.7	10.5	0.7358	–	–	–
N226A	133.0	34.0	0.6179	–	–	–
Q231A	140.6	14.8	0.2281	–	–	–
P233A	111.3	23.5	0.8777	165.5	83.5	0.1875
I235S	106.1	2.3	0.1020	105.3	4.9	0.6516
L236S	107.8	6.1	0.2990	103.6	10.7	0.8028
K240A	110.4	9.4	0.7430	104.3	29.5	0.7760
G243I	133.6	19.4	0.5610	–	–	–
D244A	80.3	21.9	0.5910	109.3	18.1	0.5394
G247I	93.3	24.8	0.7640	–	–	–
F249A	118.1	5.4	0.0566	100.1	6.5	0.7431
F249Y	104.3	3.2	0.2037	114.6	9.4	0.4105
A250R	160.3	32.4	0.2910	–	–	–
A250V	120.4	19.6	0.4320	113.1	13.2	0.1000
K254A	99.3	2.2	0.5932	–	–	–
P259A	87.8	27.6	0.6490	91.4	13.9	0.4800
D269A	79.1	6.4	0.1424	50.6	10.1	0.0082
D269E	99.6	14.0	0.8410	84.6	20.5	0.2722
D269R	15.9	5.3	0.0069	10.3	4.4	0.0001
D269A+K272A	90.0	12.4	0.8557	81.2	8.1	0.0414
D269A+K272R	6.3	1.9	0.0019	13.9	9.6	0.0001
D269A+K272D	9.1	5.9	0.0181	14.1	3.5	0.0002
D269E+K272A	29.2	8.3	0.0333	33.7	5.4	0.0010
D269R+K272A	15.6	2.3	0.3110	12.3	3.0	0.0001
D269A+R280A	4.9	2.9	0.0024	13.3	6.4	0.0001
D269A+R280K	12.6	4.5	0.2235	13.1	20.3	0.0011
D269A+R280D	17.1	14.6	0.0007	18.3	13.3	0.0001
D269E+R280K	117.5	9.6	0.2252	104.4	7.8	0.8373
D269R+R280D	23.9	8.1	0.0299	18.5	6.0	0.0001
D269E+R280D	68.3	19.1	0.4674	61.3	9.2	0.0100
D269R+R280K	20.5	3.1	0.0346	24.1	2.7	0.0005
K272A	78.1	6.8	0.2243	69.7	6.0	0.0003
K272R	104.5	4.3	0.5892	99.1	17.0	0.8670
K272D	31.7	5.4	0.0683	29.0	11.2	0.0027
K272A+R280A	11.8	2.6	0.0018	22.8	5.5	0.0001
K272A+R280K	61.5	6.5	0.1100	71.0	8.2	0.0153
K272A+R280D	77.1	13.2	0.8951	61.3	9.4	0.0231
K272R+R280K	71.4	2.5	0.2537	80.8	14.0	0.093
K272D+R280D	22.1	4.0	0.0438	17.2	4.6	0.0001
K272D+R280A	10.3	1.6	0.0096	8.7	2.3	0.0001
K272R+R280A	113.8	6.9	0.2211	123.3	13.5	0.1200
K272R+R280D	58.2	10.2	0.0425	69.7	8.5	0.0144
E273A	103.8	8.4	0.6596	99.6	18.1	0.4324
R277A	106.0	16.3	0.9064	–	–	–
D278A	106.3	21.1	0.9371	105.5	6.5	0.4636
Y279A	95.6	13.7	0.8189	91.3	19.0	0.494
Y279F	115.7	35.4	0.8857	–	–	–
R280A	100.8	4.7	0.9792	97.7	5.6	0.6706
R280K	79.6	15.0	0.2151	87.0	30.3	0.5823
R280D	86.6	4.9	0.4183	87.4	10.8	0.4947
E281A	102.3	22.2	0.9191	92.8	23.5	0.8111
E281R	145.1	50.7	0.4875	123.9	35.5	0.5364
G283V	194.7	47.0	0.2506	–	–	–
C285A	67.6	26.0	0.2984	–	–	–
R286A	90.8	6.9	0.8308	95.9	17.4	0.9604
K291A	95.4	8.2	0.2335	95.0	5.4	0.4128

Table S1. (ctd.)

F293A	108.2	20.5	0.8202	117.0	14.6	0.1891
R294A	105.6	16.7	0.2179	–	–	–
P302A+P303A	135.8	2.7	0.1050	–	–	–
P303A	125.4	12.5	0.1330	–	–	–
W308A	109.7	7.5	0.7973	103.9	14.1	0.9622
F309A	97.7	8.1	0.9266	95.2	15.6	0.6601
F309Y	105.7	5.9	0.8315	133.4	25.4	0.3589
E312A	130.1	29.3	0.7476	–	–	–
W313A	96.6	37.9	0.6040	–	–	–
F319A	81.0	33.5	0.5932	–	–	–
E321A	69.6	8.2	0.0572	87.1	19.0	0.4114
E321A+E325A	107.1	3.9	0.2250	–	–	–
E325A	110.1	15.2	0.5613	–	–	–
R328A	82.5	7.7	0.0981	–	–	–
R328K	86.5	22.6	0.5481	96.9	17.3	0.8698
Y329A	78.3	1.3	0.2806	76.0	6.1	0.1309
Y338A	99.3	11.0	0.8443	–	–	–
R340A	89.0	4.2	0.8425	–	–	–
V342A	125.4	47.4	0.6920	–	–	–
V342A+V343A	77.4	4.7	0.7620	–	–	–
P345A	102.1	19.4	0.9443	–	–	–
R355A	92.9	10.0	0.7433	110.0	18.3	0.5568
D359A	20.2	7.1	0.0401	27.0	6.4	0.0004
D359E	115.1	33.8	0.8554	73.7	15.5	0.1506
C373A	146.3	14.6	0.1955	–	–	–
D378A	108.6	19.0	0.7991	–	–	–
D381A	81.0	35.9	0.5865	–	–	–
Y387A	93.9	17.2	0.4694	–	–	–
Y387A+Y391A	73.9		0.1764	–	–	–
Y387A+Y395A	90.6	1.4	0.4163	–	–	–
S388V	106.6	6.9	0.799	93.3	10.3	0.6193
Y391A	97.3	12.0	0.6848	–	–	–
Y395A	85.3	6.8	0.5683	–	–	–
F396A	78.8	8.2	0.3325	133.3	27.1	0.2124
Y398A	67.1	1.5	0.1003	–	–	–
Y391A+Y398A	105.1	10.2	0.5682	–	–	–
W400A	105.4	35.3	0.9462	101.3	14.8	0.9127
C405A	99.0	36.6	0.9738	–	–	–

All *wzx* mutations were created in the pHERD26T-wzx-GFP-His_8 plasmid and used to complement a Δ*wzx* chromosomal knockout strain of *P. aeruginosa* PAO1. Conservative physicochemical amino acid substitutions have been indicated with an indent under their respective initial mutants to examine potential loss of function.

Red = considerable difference from native complementation, but not statistically significant (p value > 0.05)

Blue = considerable difference from native complementation, displaying statistical significance (p value ≤ 0.05)

Black = no difference from native complementation

[1]Screen performed using three independent cultures each analyzed once.

[2]Screen performed using three independent cultures, each analyzed in triplicate.

[3]Empty pHERD26T vector present in the Δ*wzx* chromosomal knockout strain of *P. aeruginosa* PAO1.

Table S2. Correlated sequence contact pairs amongst 32 Wzx proteins.

Positions (α:β)[a]	Paired Amino Acids[b]	Occurrences (out of 32)[c]	ConSurf Conservation Score (out of 9)[d]	
TMS1:TMS8			*Position* α	*Position* β
16:264	T/S:G/A/S	30	9	8
20:264	Q:G/A/S	27	9	8
28:254	L/P:K/R	23	9	8
TMS2:TMS7				
45:229	Y/F/W:G/A/S	23	7	8
45:233	Y/F/W:P	24	7	8
59:218	R/K:Y/F	21	8	6
60:215	Y/F:Y/F	23	8	7
60:218	Y/F:Y/F	25	8	6
63:218	A:Y/F	23	9	6
TMS2:TMS8				
59:263	R/K:L/I/V/F/M	26	8	7
59:264	R/K:G/A/S	26	8	8
63:271	A/S:Y/F	28	9	8
TMS3:TMS7				
76:208	L/I/V:L/I/V	21	8	4
76:215	L/I/V:Y/F/H	24	8	7
79:212	L/I/V:Y/F/H	30	8	8
79:215	L/I/V:Y/F/H	23	8	7
87:216	L/I/V/F/M:P	20	4	9

[a] Position of the amino acid as numbered according to Wzx from *P. aeruginosa* PAO1. α, first position; β, second position

[b] Identified via multiple sequence alignment (MSA) of Wzx from *P. aeruginosa* PAO1 with 31 closest full-length BLASTp hits.

[c] Number of times a given pair of physicochemically-interchangeable amino acids appeared at conserved positions in the MSA.

[d] ConSurf conservation color score (out of 9) of amino acid positions in the structural model of Wzx from *P. aeruginosa* PAO1 based on the 32-protein Wzx MSA (Landau *et al*, 2005). For comparison, the ConSurf score for each of the eight functionally-important residues identified (Fig. 1) is as follows (scores in brackets): Arg59 (8), Tyr60 (8), Glu61 (8), Phe139 (8), Arg146 (7), Asp269 (8), Lys272 (5), Asp359 (2).

Table S3. Normalized activities of AP and BG for Wzx truncation fusions to PhoALacZα

Residue[a]	Avg AP[b]	Avg BG[c]	%AP[d]	%BG[e]	NAR[f] (%AP÷%BG)	Localization[g]
Random						
T27	147.6	19.4	31.8	18.9	1.68	Core TMS1
R32	464.8	9.1	100.0	8.9	11.25	Core TMS1
S43	459.4	0.2	98.8	0.2	>100	Peripheral periplasmic TMS2 cap
F65	-1.6	19.1	-0.3	18.7	<0.01	Cytoplasmic loop 1 (i.e. flanked by TMS2 and TMS3)
G71	-3.2	16.1	-0.7	15.7	<0.01	Cytoplasmic loop 1 (i.e. flanked by TMS2 and TMS3)
I80	0.0	49.2	0.0	48.0	<0.01	Peripheral cytoplasmic TMS3 cap
H100	113.7	0.2	24.5	0.2	>100	Peripheral periplasmic TMS3 cap
V158	168.1	45	36.2	43.9	0.82	Core TMS5
N196	-2.6	41.3	-0.6	40.3	<0.01	Cytoplasmic loop 3 (i.e. flanked by TMS6 and TMS7)
C197[†]	-0.8	34.1	-0.2	33.3	<0.01	Cytoplasmic loop 3 (i.e. flanked by TMS6 and TMS7)
W202	10.4	95.3	2.2	93.1	0.02	Cytoplasmic loop 3 (i.e. flanked by TMS6 and TMS7)
R203	2.5	48.9	0.5	47.8	0.01	Cytoplasmic loop 3 (i.e. flanked by TMS6 and TMS7)
V208	0.5	47.3	0.1	46.2	<0.01	Cytoplasmic terminus of TMS7
P221[†]	2.1	102.4	0.5	100.0	0.01	Core TMS7
S245[†]	1.2	31.4	0.3	30.7	0.01	Peripheral periplasmic TMS8 cap
I255[†]	18.4	86	4.0	84.0	0.05	Core TMS8
G287	-4.5	29.6	-1.0	28.9	<0.01	Peripheral cytoplasmic TMS9 cap
Targeted						
W113	8	-4.4	1.6	-0.8	>100	Peripheral periplasmic TMS4 cap
S138	-3.9	10.8	-0.8	2.0	<0.01	Cytoplasmic terminus of core TMS4
S168	156.8	-5.3	31.9	-1.0	>100	Periplasmic loop 3 (i.e. flanked by TMS5 and TMS6)
V178	491.3	28.9	100.0	5.4	18.49	Core TMS6
Y212	-6.1	34.5	-1.2	6.5	<0.01	Peripheral cytoplasmic TMS7 cap
L251	7.9	37.7	1.6	7.1	0.23	Core TMS8
D269	25.9	15.4	5.3	2.9	1.83	Peripheral cytoplasmic TMS8 cap
G283[†]	-3.1	62.9	-0.6	11.8	<0.01	Cytoplasmic terminus of TMS9
F304	46.2	12.8	9.4	2.4	3.93	Core TMS9
G311	13.8	-7.4	2.8	-1.4	>100	Peripheral periplasmic TMS9 cap
V318	22.9	54.5	4.7	10.2	0.46	Periplasmic loop 5 (i.e. flanked by TMS9 and TMS10)
E325	5.9	0.9	1.2	0.2	7.13	Peripheral periplasmic TMS10 cap
F374	6.3	-2.4	1.3	-0.4	>100	Peripheral periplasmic TMS11 cap
D381	158.3	-1.9	32.2	-0.4	>100	Peripheral periplasmic TMS12 cap
S402	-2.8	2.5	-0.6	0.5	<0.01	Cytoplasmic terminus of core TMS12
K411	-2.9	18.4	-0.6	3.4	<0.01	Cytoplasmic C-terminal tail of Wzx

As purpose-built truncations had higher absolute activities than random truncations, the two sets were normalized independently against the maximum activity within the respective subset of residues. This difference in absolute activities between exonuclease III (randomly)-generated constructs and those that were directly cloned (targeted) was attributed to the linker between the C-terminal truncation of the latter set of clones and the dual reporter being spaced two amino acids further away, resulting in fewer steric constraints. These additional two amino acid residues were the result of the 3' PstI endonuclease restriction site used for cloning of the given truncated construct upstream of the *phoAlacZα* moiety (Islam *et al*, 2010).

[a] Position of the terminal amino acid of Wzx followed by reporter.

[b] and [c] AP and BG activities of the fusions in Miller units, measured as described in Materials and Methods; average of four independent experiments.

[d] and [e] Percentage of AP and BG activities of the fusions in relation to the maximum measured activity within the set, calculated separately for targeted and random truncations.

[f] Normalized %AP ÷ %BG activity ratio (NAR), rounded to two decimal places.

[g] Localization of terminal amino acid in the Wzx homology model based on colouring via iMembrane output (Fig. 4)

[†] AP and BG enzyme activity values were obtained for this investigation. Values for all other listed residues were determined previously (Islam *et al*, 2010)

Table S4. Oligonucleotide primers used to generate site-directed mutants of Wzx-GFP-His$_8$

Mutation	Sense	DNA Sequence (5'→3')
R11A	Forward	CCGTGTCGCAGCAGTATTAGTAGGC
	Reverse	GCCTACTAATACTGCTGCGACACGG
R32A	Forward	CTGCTACTGGTTGCTCTGTATTCTCC
	Reverse	GGAGAATACAGAGCAACCAGTAGCAG
P36A	Forward	CTGTATTCTGCTGCTGAAATG
	Reverse	CATTTCAGCAGCAGAATACAG
E38A	Forward	CCTGCTGCAATGGGCG
	Reverse	CGCCCATTGCAGCAGG
F42A	Forward	GGGCGCTGCCAGTGTTTG
	Reverse	CAAACACTGGCAGCGCCC
G58V	Forward	GTAGTTACTGTGCGCTATGAGTTG
	Reverse	CCAACTCATAGCGCACAGTAACTAC
R59A	Forward	GTAGTTACTGGGGCCTATGAGTTGG
	Reverse	CCAACTCATAGGCCCCAGTAACTAC
R59K	Forward	GTTGTAGTTACTGGGAAATATGAGTTGGC
	Reverse	GCCAACTCATATTTCCCAGTAACTACAAC
Y60A	Forward	CTGGGCGCTTTGAGTTGGC
	Reverse	GCCAACTCAAAGCGCCCAG
Y60F	Forward	CTGGGCGCTATGCGTTGGC
	Reverse	CGAAAAAAATAGCCAACGCATAGCG
E61A	Forward	GGCGCTATGATTTGGCTATTTTTTC
	Reverse	GAAAAAATAGCCAAATCATAGCGCC
E61D	Forward	GAGTTGGCTGCTTTTTCGACTCGAG
	Reverse	GTCGAAAAAGCAGCCAACTCATAGC
I64A	Forward	CTATTTTTTCGGCTCGAGAAGAG
	Reverse	CTCTTCTCGAGCCGAAAAAATAG
T67A	Forward	CGACTGCAGAAGAGGGCG
	Reverse	CTCTTCTGCAGTCGAAAAAATAGC
R68A	Forward	CGAGCAGAGGGCGAACTCC
	Reverse	CTCTGCTCGAGTCGAAAAAATAGC
E69A	Forward	CTGGGCGCTTTGAGTTGGC
	Reverse	GCCAACTCAAAGCGCCCAG
E69A + E70A	Forward	CGAGCAGCGGGCGAACTCCAG
	Reverse	GCCCGCTGCTCGAGTCGAAAAAATAG
E70A	Forward	GAAGCGGGCGAACTCCAGG
	Reverse	CGCCCGCTTCTCGAGTCG
E72A	Forward	GAGGGCGCACTCCAGGC
	Reverse	GCCTGGAGTGCGCCCTC
K78A	Forward	GGCAATCGTCGCGCTGATACTTCAG
	Reverse	CTGAAGTATCAGCGCGACGATTGCC
K78R	Forward	CAATCGTCAGGCTGATAC
	Reverse	GTATCAGCCTGACGATTG
I87A	Forward	GACACTATTGGCTTTCGTTGCC
	Reverse	GGCAACGAAAGCCAATAGTGTC
F88A	Forward	CACTATTGATTGCCGTTGCCGTGG
	Reverse	CCACGGCAACGGCAATCAATAGTG
V91A	Forward	GTTGCCGCGGCGATTGC
	Reverse	GCAATCGCCGCGGCAAC
R99A	Forward	TGGCGATTGCTGTTGTTATAGGTGCACATCTGATTGAGTCG
	Reverse	CGACTCAATCAGATGTGCACCTATAACAACAGCAATCGCCA
H100A	Forward	GGCGATTGCTGTTGTTATAGGTAGAGCTCTGATTGAGTCG
	Reverse	CGACTCAATCAGAGCTCTACCTATAACAACAGCAATCGCC
E103A	Forward	CTGATTGCGTCGATGCCAG
	Reverse	CTGGCATCGACGCAATCAG

Table S4. (ctd.)

G110V	Forward	GTTGTGATCGTTGAATACTGGTTC
	Reverse	GAACCAGTATTCAACGATCACAAC
E111A	Forward	GTGATCGGTGCATACTGGTTCG
	Reverse	CGAACCAGTATGCACCGATCAC
W113A	Forward	CGGTGAATACGCGTTCGCATTGG
	Reverse	CCAATGCGAACGCGTATTCACCG
A115V	Forward	CTGGTTCGTATTGGCGGTGG
	Reverse	CCACCGCCAATACGAACCAG
K127A	Forward	CGACAAGACTAGCGCATTTATCCCC
	Reverse	GGGGATAAATGCGCTAGTCTTGTCG
F139A	Forward	CAACAATCTGCTAATCGGTTGGGAG
	Reverse	CTCCCAACCGATTAGCAGATTGTTG
F139Y	Forward	CAACAATCTTATAATCGGTTGGG
	Reverse	CCCAACCGATTATAAGATTGTTG
N140A	Forward	CAATCTTTTGCTCGGTTGGGAG
	Reverse	CTCCCAACCGAGCAAAAGATTG
R141A	Forward	CAACAATCTTTTAATGCGTTGGGAGTTGC
	Reverse	GCAACTCCCAACGCATTAAAAGATTGTTG
R146A	Forward	CCAGGCTTACAGCAGCAACTCCC
	Reverse	GGGAGTTGCTGCTGTAAGCCTGG
R146K	Forward	GTTGGGAGTTGCTAAAGTAAGCCTGGC
	Reverse	GCCAGGCTTACTTTAGCAACTCCCAAC
Y162A	Forward	CAGCTGCAGCTTTACTGGAGGGC
	Reverse	GCCCTCCAGTAAAGCTGCAGCTG
E165A	Forward	CATATTTACTGGCGGGCGTATCAGG
	Reverse	CCTGATACGCCCGCCAGTAAATATG
V180A	Forward	GTGTCGTCGCAGCCACG
	Reverse	CGTGGCTGCGACGACAC
K191A	Forward	GGGTAGGAGCGTCGCTGATTTTAAATTG
	Reverse	CAATTTAAAATCAGCGACGCTCCTACCC
N196A	Forward	CTGATTTTAGCTTGTATCGAGAC
	Reverse	GTCTCGATACAAGCTAAAATCAG
E199A	Forward	GTATCGCGACACCGTGGCG
	Reverse	GGTGTCGCGATACAATTTAAAATCAG
P201A	Forward	GAGACAGCGTGGCGTATG
	Reverse	CATACGCCACGCTGTCTC
R203A	Forward	CCGTGGGCTATGGTACGACAAGTAG
	Reverse	GTACCATAGCCCACGGTGTCTCG
R206A	Forward	GCGTATGGTAGCACAAGTAGCGG
	Reverse	CCGCTACTTGTGCTACCATACGC
R206K	Forward	GCGTATGGTAAAACAAGTAGCGG
	Reverse	CCGCTACTTGTTTTACCATACGC
F215A	Forward	GTACATCAATGCCCCGAAGTTTTCTC
	Reverse	GAGAAAACTTCGGGGCATTGATGTAC
P216A	Forward	GTACATCAATTTCGCGAAGTTTTCTC
	Reverse	GAGAAAACTTCGCGAAATTGATGTAC
K217A	Forward	CATCAATTTCCCGGCGTTTTCTCTG
	Reverse	CAGAGAAAACGCCGGGAAATTGATG
P221A	Forward	GTTTTCTCTGGCTGCGGATCTG
	Reverse	CAGATCCGCAGCCAGAGAAAAC
A222S	Forward	CTCTGCCTTCGGATCTGGTC
	Reverse	GACCAGATCCGAAGGCAGAG
D223A	Forward	CCTGCGGCTCTGGTC
	Reverse	GACCAGAGCCGCAGG

Table S4. (ctd.)

N226A	Forward	GGATCTGGTCGCCACGGTTGC
	Reverse	GCAACCGTGGCGACCAGATCC
Q231A	Forward	GTTGCCAGTGCGGTGCCTGTG
	Reverse	CACAGGCACCGCACTGGCAAC
P233A	Forward	GTCAGGTGGCTGTGATTTTATTGG
	Reverse	CCAATAAAATCACAGCCACCTGAC
I235S	Forward	CTGTGAGTTTATTGGCGGCAAAG
	Reverse	CCAATAAACTCACAGGCACCTG
L236S	Forward	CCTGTGATTTCATTGGCGGCAAAG
	Reverse	CTTTGCCGCCAATGAAATCACAGG
K240A	Forward	GGCGGCAGCGTTTGGTGGAGAC
	Reverse	CCAAACGCTGCCGCCAATAAAATCAC
G243I	Forward	CAAAGTTTGGTATAGACAGTGCAG
	Reverse	CTGCACTGTCTATACCAAACTTTG
D244A	Forward	GAGCCAGTGCAGGCTGGTTTG
	Reverse	CTGGCTCCACCAAACTTTGCCG
G247I	Forward	GACAGTGCAATCTGGTTTGCCC
	Reverse	GGGCAAACCAGATTGCACTGTC
F249A	Forward	CTGGGCTGCCCTGACTCTGAAG
	Reverse	CAGCCCAGCCTGCACTGTCTC
F249Y	Forward	CTGGTATGCCCTGACTCTGAA
	Reverse	CATACCAGCCTGCACTGTCTC
A250R	Forward	GGTTTCGCCTGACTCTGAAGATAATG
	Reverse	GCGAAACCAGCCTGCACTGTCTC
A250V	Forward	GGAGACAGTGTAGGCTGGTTTG
	Reverse	CAAACCAGCCTACACTGTCTCC
K254A	Forward	CCCTGACTCTGGCGATAATGGGAG
	Reverse	CTCCCATTATCGCCAGAGTCAGGG
P259A	Forward	GGGAGCTGCCATTTCCTTGTTG
	Reverse	CAACAAGGAAATGGCAGCTCCC
D269A	Forward	CGGTGCTCGCTGTGTTCAAAG
	Reverse	CTTTGAACACAGCGAGCACCG
D269E	Forward	CGGTGCTCGAAGTGTTCAAAG
	Reverse	CTTTGAACACTTCGAGCACCG
D269R	Forward	GGTGCTCCGTGTGTTCAAAGAAC
	Reverse	CACACGGAGCACCGAAGCAG
K272A	Forward	GTTCGCAGAACAAGCCGCTCGTG
	Reverse	GTTCTGCGAACACATCGAGCACCG
K272R	Forward	CAGAGAACAAGCCGCTCGTG
	Reverse	GTTCTCTGAACACATCGAGCAC
K272D	Forward	GATGTGTTCGATGAACAAGCCGCTC
	Reverse	GAGCGGCTTGTTCATCGAACACATC
E273A	Forward	GAGCGGCTTGTGCTTTGAACAC
	Reverse	GTGTTCAAAGCACAAGCCGCTC
R277A	Forward	CGGTAGTCAGCAGCGGCTTGTTC
	Reverse	GAACAAGCCGCTGCTGACTACCG
D278A	Forward	GGTAGGCACGAGCGGCTTG
	Reverse	CGTGCCTACCGAGAGTTTGG
Y279A	Forward	CTCGTGACGCCCGAGAGTTTG
	Reverse	CAAACTCTCGGGCGTCACGAG
Y279F	Forward	CGTGACTTCCGAGAGTTTGG
	Reverse	CGGAAGTCACGAGCGGC
R280A	Forward	CCAAACTCTGCGTAGTCACGAGCG
	Reverse	CGCTCGTGACTACGCAGAGTTTGG

Table S4. (ctd.)

R280K	Forward	GCTCGTGACTACAAAGAGTTTGGTAATTG
	Reverse	CAATTACCAAACTCTTTGTAGTCACGAGC
R280D	Forward	CGTGACTACGATGAGTTTGGTAATTGCCG
	Reverse	CGGCAATTACCAAACTCATCGTAGTCACG
E281A	Forward	GCGGTAGTCACGAGCGGC
	Reverse	CGGCAATTACCAAACGCTCGG
E281R	Forward	GACTACCGACGGTTTGGTAATTGCC
	Reverse	CGGCAATTACCAAACCGTCGGTAGTC
G283V	Forward	CCGAGAGTTTGTTAATTGCCGAG
	Reverse	CTCGGCAATTAACAAACTCTCGG
C285A	Forward	GGTAATGCCCGAGGTATC
	Reverse	GATACCTCGGGCATTACC
R286A	Forward	GTTTGGTAATTGCGCAGGTATCTTCCTC
	Reverse	GAGGAAGATACCTGCGCAATTACCAAAC
K291A	Forward	GTATCTTCCTCGCGACTTTCAGGTTG
	Reverse	GCAACCTGAAAGTCGCGAGGAAGATAC
F293A	Forward	GACTGCCAGGTTGCTTGCCGTC
	Reverse	GCAACCTGGCAGTCTTGAGGAAG
R294A	Forward	CTCAAGACTTTCGCGTTGCTTGCCG
	Reverse	CGGCAAGCAACGCGAAAGTCTTGAG
P302A + P303A	Forward	GCGCTAGCTGCTTTTATTATATTTTGGTTC
	Reverse	GCAGCTAGCGCGAGGACGGCAAGC
W308A	Forward	CCTTTTATTATATTTGCGTTCATTGGCGAG
	Reverse	CTCGCCAATGAACGCAAATATAATAAAAGG
F309A	Forward	CTTTTATTATATTTTGGGCCATTGGCGAG
	Reverse	CTCGCCAATGGCCCAAAATATAATAAAAG
F309Y	Forward	CTTTTATTATATATTGGTTCATTGGC
	Reverse	GCCAATGAACCAATATATAATAAAAG
E312A	Forward	CGCGTGGGCCTTTGGGTTAG
	Reverse	GCCCACGCGCCAATGAACC
W313A	Forward	GCGAGGCGGCCTTTG
	Reverse	CAAAGGCCGCCTCGC
F319A	Forward	CTTTGGGTTAGTCGCTGGCGAAG
	Reverse	CTTCGCCAGCGACTAACCCAAAG
E321A	Forward	GTCTTTGGCGCAGCGTGGGC
	Reverse	GCCCACGCTGCGCCAAAGAC
E325A	Forward	GTGGGCTGCGTCGGGGC
	Reverse	GCCCCGACGCAGCCCAC
R328A	Forward	CAATACAGCATAGCGCCCCGACTCAG
	Reverse	CTGAGTCGGGGCGCTATGCTGTATTG
R328K	Forward	GAGTCGGGGAAATATGCTGTATTG
	Reverse	CAATACAGCATATTTCCCCGACTC
Y329A	Forward	GGCGTGCTGCTGTATTGATGG
	Reverse	CAGCAGCACGCCCCGAC
Y338A	Forward	GTTGTTTGCTATGCGTTTCGTGG
	Reverse	CGCATAGCAAACAACGGAACC
R340A	Forward	GTTGTTTTATATGGCTTTCGTGGTGAG
	Reverse	CTCACCACGAAAGCCATATAAAACAAC
V342A[†]	Forward	CGGCGAGTCCGCTCAGCTATAC
	Reverse	CGCCGCGAAACGCATATAAAACAAC
V342A + V343A	Forward	CGGCGAGTCCGCTCAGCTATAC
	Reverse	CGCCGCGAAACGCATATAAAACAAC
P345A	Forward	GGTGAGTGCGCTCAGC
	Reverse	GCTGAGCGCACTCACC

Table S4. (ctd.)

R355A	Forward	CTATATTGCCCAG**GC**GCAGAGTATGG
	Reverse	CCATACTCTGC**GC**CTGGGCAATATAG
D359A	Forward	GCAGAGTATGG**C**TTTGTTGTGGC
	Reverse	GCCACAACAAA**G**CCATACTCTGC
D359E	Forward	CAGAGTATGGA**A**TTGTTGTGGC
	Reverse	GCCACAACAA**T**TCCATACTCTG
C373A	Forward	GTTTATC**GC**TTTTACCTTGCCTG
	Reverse	GTAAAA**GC**GATAAACGTCAGGAG
D378A	Forward	CTTGCCTG**C**CTCTGTCG
	Reverse	CGACAGAG**G**CAGGCAAG
D381A	Forward	GACTCTGTCG**C**CTCGGTGTTG
	Reverse	CAACACCGAG**G**CGACAGAGTC
Y387A	Forward	TGTCGACTCGGTGTTGTGGTTT**GC**CTCCATAGCATATGCTG
	Reverse	GGAG**GC**AAACCACAACACC
S388V	Forward	GTGGTTTTAC**GT**CATAGCATATGCTG
	Reverse	CAGCATATGCTATG**AC**GTAAAACCAC
Y391A	Forward	CCATAGCA**GC**TGCTGTTATG
	Reverse	CATAACAGCA**GC**TGCTATGG
Y395A	Forward	GCTGTTATG**GC**TTTTGTCTATTTC
	Reverse	GAAATAGACAAAA**GC**CATAACAGC
F396A	Forward	GTTATGTAT**GC**TGTCTATTTCTGG
	Reverse	GAAATAGACA**GC**ATACATAACAGC
Y398A	Forward	GTATTTTGTCGCTTTCTGGATG
	Reverse	CATCCAGAAAGCGACAAAATAC
W400A	Forward	GTCTATTTC**GC**GATGTCCTTCCAGTG
	Reverse	CACTGGAAGGACATC**GC**GAAATAGAC
C405A	Forward	CTTCCAG**GC**TGCCAAGG
	Reverse	CCTTGGCA**GC**CTGGAAG

All *wzx* mutations were created in the pHERD26T-wzx-GFP-His_8 plasmid (Islam *et al.*, 2010).

Bold Red = nucleotide mismatch designed to introduce the desired mutation in the template construct.

[†]The same primers used to generate the V342A + V343A double mutant also yielded a reaction in which only the V342A mutation was introduced, resulting in the creation of the single V342A mutant.

SUPPORTING INFORMATION REFERENCES

Chen, V.B., Arendall, W.B., Headd, J.J., and Keedy, D.A., Immormino, R.M., Kapral, G.J., Murray, L.W., Richardson, J.S., and Richardson, D.C. (2010) MolProbity: all-atom structure validation for macromolecular crystallography. *Acta Crystallogr D Biol Crystallogr* **66:**12-21.

He, X., Szewczyk, P., Karyakin, A., Evin, M., Hong, W.X., Zhang, Q., and Chang, G. (2010) Structure of a cation-bound multidrug and toxic compound extrusion transporter. *Nature* **21:**991-994.

Ho, B.K. and Gruswitz, F. (2008) HOLLOW: Generating accurate representations of channel and interior surfaces in molecular structures. *BMC Struct Biol* **8:**49.

Hvorup, R.N., Winnen, B., Chang, A.B., Jiang, Y., Zhou, X.-F., and Saier Jr, M.H. (2003) The multidrug/oligosaccharidyl-lipid/polysaccharide (MOP) exporter superfamily. *Eur J Biochem* **270:**799-813.

Islam, S.T., Gold, A.C., Taylor, V.L., Anderson, E.M., Ford, R.C., and Lam, J.S. (2011) Dual conserved periplasmic loops possess essential charge characteristics that support a catch-and-release mechanism of O-antigen polymerization by Wzy in *Pseudomonas aeruginosa* PAO1. *J Biol Chem* **286:**20600-20605.

Islam, S.T., Taylor,V.L., Qi, M., and Lam, J.S. (2010) Membrane topology mapping of the O-antigen flippase (Wzx), polymerase (Wzy), and ligase (WaaL) from *Pseudomonas aeruginosa* PAO1 reveals novel domain architectures. *mBio* **1:**e00189-10.

Khafizov, K., Staritzbichler, R., Stamm, M., and Forrest, L.R. (2010) A study of the evolution of inverted-topology repeats from LeuT-fold transporters using AlignMe. *Biochemistry* **49:**10702-10713.

Landau, M., Mayrose, I., Rosenberg, Y., Glaser, F., Martz, E., Pupko, T., and Ben-Tal, N. (2005) ConSurf 2005: the projection of evolutionary conservation scores of residues on protein structures. *Nucl Acids Res* **33:**W299-W302.

Pawlowski, M., Gajda, M., Matlak, R., and Bujnicki, J. (2008) MetaMQAP: A meta-server for the quality assessment of protein models. *BMC Bioinformatics* **9:**403.

Waterhouse, A.M., Procter, J.B., Martin, D.M.A., Clamp, M., and Barton, G.J. (2009) Jalview Version 2—a multiple sequence alignment editor and analysis workbench. *Bioinformatics* **25:**1189-1191.
